# The complete mitogenome of *Gracilaria chouae* and its phylogenetic analysis

**DOI:** 10.1080/23802359.2019.1659113

**Published:** 2019-08-29

**Authors:** Xianming Tang, Xuli Jia, Jing Zhang, Tao Liu

**Affiliations:** a Hainan Academy of Ocean and Fisheries Sciences, Haikou, People's Republic of China;; b Hainan Provincial Key Laboratory of Technology for Tropical Seawater Aquaculture, Haikou, People's Republic of China;; c Laboratory of Genetics and Breeding of Marine Organism, College of Marine Life Sciences, Ocean University of China, Qingdao, People's Republic of China;; d Qi Lu University of Technology (Shandong Academy of Sciences), Jinan, People's Republic of China

**Keywords:** *Gracilaria chouae*, mitogenome, phylogenetic analysis, Gracilariaceae

## Abstract

*Gracilaria chouae*, a marine red macroalgae, is a rich source of active substances and is listed as biological and health food material with high economic value. The mitogenome sequence of *G. chouae* is 25,829 bp. A total of 50 genes were determined, including 24 protein-encoding genes, two rRNA genes, 23 tRNA genes, and one unidentified open reading frame (ORF). Phylogenetic analysis showed that *G. chouae* clustered together with *Gracilariopsis chorda*, *Gracilariopsis lemanaeformis*, *Gracilariopsis andersonii*, *Gracilariophila oryzoides*, and *Gracilariopsis heteroclada.* The mitogenome analysis will help the understanding of Gracilaria evolution.

*Gracilaria chouae* Zhang et Xia is the indigenous species in Shandong and Fujian Province and south of China. It usually grows on gravel or shells in lower littoral tide pools or in the subtropics zone (Xu et al. 2014). It commercially important algae due to intensive culture, rapid growth, and high yields. A few studies on this species focus on the improvement of culture techniques (Zhou et al. 2016) and transcriptomic analysis (Xu et al. 2014). In addition, the extraction of polysaccharides from *G. chouae* could induce apoptosis of cancer cells (Yaoyao et al. 2016). However, genomic studies on this species are relatively limited.

Herein, we determined the complete *G. chouae* mitogenome sequence. Genomic DNA from one *G. chouae* individual collected from a population in eastern China (Shantou, Guangdong Province, 23°25′10′′N, 117°1′2′′E) was used for genome sequencing. The specimen (sample accession number: 2016060196) was deposited at the Culture Collection of Seaweed at the Ocean University of China. Paired-end reads were sequenced using Illumina HiSeq × Ten system (Illumina, USA). Approximately 9 Gb of paired-end (150 bp) sequence data were randomly extracted from the total sequencing output, as input to NOVOPlasty (Dierckxsens et al. 2017) for assembling the mitogenome. *Gracilaria salicornia* (GenBank accession number: NC_023784) was used as the seed sequence. tRNA genes were identified using tRNAscan-SE Search Server (Schattner et al. 2005). Other mitogenomic regions were annotated from *G. salicornia* mitogenome using Geneious R10 (Biomatters Ltd, Auckland, New Zealand). Phylogenetic analysis was conducted using MrBayes v. 3.1.2 (Huelsenbeck and Ronquist 2001), it was carried out using two independent runs with four Monte-Carlo Markov Chains running for 1,000,000 generations, Output trees were sampled every 100 generations. The phylogenetic analysis was run until the average standard deviation of split frequencies was below 0.01, and the first 25% of samples was removed as burn-in. *Rhodymenia pseudopalmata* (KC875852) and *Sebdenia flabellata* (KJ398164) served as the out-group.

The complete *G. chouae* (MG733298) mitogenome comprises a circular DNA molecule measuring 25,829 bp in length. The overall A + T content of the complete mitogenome is 71.2%. The mitogenome contains 50 genes, including 24 protein-coding, two rRNA, 23 tRNA genes, and one unidentified open reading frame (ORF). Of the 24 protein-coding genes, 21 (87.5%) ended with the TAA stop codon, and three (12.5%) with TAG (*atp*8, *rps*11, and *nad*4L). All protein-coding genes in *G. chouae* were concluded to use the start codon ATG. The lengths of two rRNA genes are 2626 bp (*LSU* rRNA) and 1399 bp (*SSU* rRNA). The gene numbers and structures were largely similar among Gracilariaceae species published in the NCBI sequence database.

Phylogenetic analysis based on 20 shared mitochondrial coding protein sequences from 15 red algal mitogenomes. All red algal taxa were clearly separated according to their original class (Figure 1). Gracilariaceae species formed a large branch, in which *G. chouae* formed a sub-branch. The complete mitogenome sequence provided herein would help understand Gracilaria evolution.

**Figure 1.**
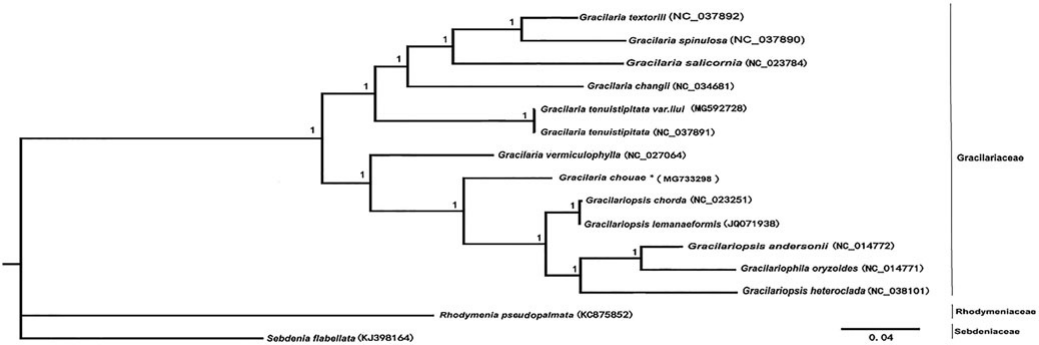
Phylogenetic tree (Bayesian inference) based on complete mitogenomes of Gracilariaceae. Support values for each node were calculated from Bayesian posterior probability (BPP). Asterisks following species names indicate newly determined mitogenomes.

## References

[CIT0001] Dierckxsens N, Mardulyn P, Smits G 2017 NOVOPlasty: de novo assembly of organelle genomes from whole genome data. Nucleic Acids Res. 45:e18 28204566 10.1093/nar/gkw955 PMC5389512

[CIT0002] Huelsenbeck JP, Ronquist F 2001 MRBAYES: Bayesian inference of phylogenetic trees. Bioinformatics (Oxford, England). 17:754–755. 10.1093/bioinformatics/17.8.754 11524383

[CIT0003] Schattner P, Brooks AN, Lowe TM 2005 The tRNAscan-SE, snoscan and snoGPS web servers for the detection of tRNAs and snoRNAs. Nucleic Acids Res. 33:W686–W689. 15980563 10.1093/nar/gki366 PMC1160127

[CIT0004] Xu J, Sun J, Yin J, Wang L, Wang X, Liu T, Chi S, Liu C, Ren L, Wu S, et al. 2014 Comparative analysis of four essential Gracilariaceae species in China based on whole transcriptomic sequencing. Acta Oceanol Sin. 33:54–62.

[CIT0005] Yaoyao JU, Cao C, Chen M, Tianwen YE, 2016 Optimization of extraction of polysaccharides from Gracilaria chouae and their inhibitory effects on tumor cells. Food Sci. 37:57–62.

[CIT0006] Zhou W, Sui Z, Wang J, Hu Y, Kang KH, Hong HR, Niaz Z, Wei H, Du Q, Peng C, et al. 2016 Effects of sodium bicarbonate concentration on growth, photosynthesis, and carbonic anhydrase activity of macroalgae *Gracilariopsis lemaneiformis*, *Gracilaria vermiculophylla*, and *Gracilaria chouae* (Gracilariales, Rhodophyta). Photosynth Res. 128:259–270. 26960545 10.1007/s11120-016-0240-3

